# Apolipoprotein-J blocks increased cell injury elicited by ox-LDL via inhibiting ROS-CaMKII pathway

**DOI:** 10.1186/s12944-019-1066-8

**Published:** 2019-05-22

**Authors:** Yanzhuo Ma, Zhi Gong, Kai Nan, Shuying Qi, Yu Chen, Chao Ding, Dongmei Wang, Leisheng Ru

**Affiliations:** 1 Department of Cardiology, Bethune International Peace Hospital, 398, Zhongshan Road, Shijiazhuang, 050082 Hebei China; 2 Health and Medical Development Research Center of Hebei Province, Shijiazhuang, Hebei China

**Keywords:** Ox-LDL, Apolipoprotein-J, Apoptosis, Neonatal rat ventricular cells

## Abstract

**Background:**

Oxidized low-density lipoprotein (ox-LDL) is crucial in cardiac injury. Apolipoprotein-J (ApoJ) contributes to antiapoptotic effects in the heart. We aimed to evaluate the protective effects of ApoJ against ox-LDL cytotoxicity in Neonatal rat ventricular cells (NRVCs).

**Methods and results:**

NRVCs were damaged by exposure to ox-LDL, as shown by increased caspase-3/7 activity, enhanced caspase-3 expression, and decreased cell viability. ApoJ overexpression, using an adenovirus vector, significantly reduced ox-LDL-induced cell injury. ApoJ also prevented ox-LDL from augmenting reactive oxygen species (ROS) production, as demonstrated by elevated Nox2/gp91^phox^ and P47 expression. Furthermore, ApoJ overexpression reduced CaMKIIδ expression elicited by ox-LDL in cultured NRVCs. Upregulating CaMKIIδ activity, mediated by ox-LDL, was significantly inhibited by ApoJ overexpression. A CaMKIIδ inhibitor, KN93, prevented ApoJ’s protective effect against ox-LDL cytotoxicity. A ROS scavenger, Mn (III)meso-tetrakis (4-benzoic acid) porphyrin (Mn (III)TBAP), also attenuated CaMKIIδ’s increased expression and activity, induced by ox-LDL, and showed similar results to ApoJ by attenuating ox-LDL-induced cell damage, as ApoJ did.

**Conclusions:**

ApoJ confers cytoprotection to NRVCs against ox-LDL cytotoxicity through the ROS-CaMKII pathways.

## Background

Apolipoprotein-J (ApoJ) is a multifunctional glycoprotein widely present in tissues and body fluids. ApoJ expression is upregulated in acute myocardial infarction, atherosclerosis, myocarditis, oxidative stress, inflammation, and after injury in general [1]. ApoJ’s function is thought to limit tissue injury and/or promote tissue remodeling.

ApoJ has cytoprotective properties [2, 3], and its overexpression benefits injured blood vessels by inhibiting migration, adhesion, and proliferation of smooth muscle cells. In addition, blocking secreted ApoJ leads to increased apoptosis of neuroblastoma cells induced by the chemotherapeutic drug doxorubicin [4]. In the heart, ApoJ produces cardioprotective effects on ischemically challenged H9C2 cells and isolated adult ventricular rat cardiomyocytes after ischemia-induced death [5]. Furthermore, ApoJ-deficient mice show more impaired cardiac function and worse myocardial scarring compared to wild-type mice. Thus, ApoJ may play a critical role in reducing injury to both normal and diseased cells.

Oxidized LDL (ox-LDL) is an independent risk factor in cardiovascular heart disease (CHD), which has various deleterious cellular effects such as intracellular calcium-content alteration, necrosis, and apoptosis. In the present study, we aimed to determine whether ApoJ overexpression contributes to antiapoptotic effects in the heart against ox-LDL-induced cell injury and to clarify the underlying mechanisms.

## Methods

### Animals

Neonatal Sprague-Dawley (SD) rats were obtained from the Department of Experimental Animals of Hebei University (Shijiazhuang, Hebei, China).

### Myocyte isolation and cell culture

Single ventricular myocytes were obtained by enzymatic dissociation using collagenase (type II) and pancreatin, as described previously, albeit with some modifications [6]. We did not use the discontinuous Percoll gradient, and the differential adhesion time was 1.5 h instead of overnight. Ventricular myocardial cells from neonatal SD rats (aged 1 d; Hebei Medical University) were homogenized and dissociated with collagenase II and pancreatin six times, for 20 min at each time point. The first cell suspension was discarded to remove other cells, such as erythrocytes and fibroblasts, while the rest of the cell suspensions were washed with FBS, mixed in 10-cm plates, and incubated for 1.5 h, allowing cardiomyocyte enrichment by differential adhesion. The supernatant was then plated onto a new dish with DMEM containing 10% FBS at 37 °C. Neonatal rat ventricular cells (NRVCs) were then given different treatments. Ox-LDL was purchased from Solarbio Company (Beijing, China) and oxidized using copper sulfate. LDL oxidation was performed by incubating it with Cu_2_SO_4_ (oxidant) in PBS at 37 °C for 20 h. The oxidation process was terminated by adding excess EDTA-Na_2_. Each lot was analyzed, using agarose gel electrophoresis, for migration against LDL. This lot of ox-LDL migrated two-fold further than the native LDL.

### Adenoviral infection of NRVCs

ApoJ-expressing adenovirus was generated using the pAD-ApoJ-IRES-EGFP adenoviral vector. Adenovirus generated from pAD-IRES-EGFP was used as control, and the adenoviral vectors were synthesized using the Gateway Cloning System (Invitrogen). Isolated NRVCs were infected with the indicated adenoviruses at a multiplicity of infection of 200 with serum-free DMEM. After 4 h, the supernatant was removed and replaced with DMEM containing 10% FBS. After 36 h, the supernatant was again removed, and the NRVCs were given different treatments.

### 3-(4,5-Dimethylthiazol-2-yl)-2,5-diphenyltetrazolium bromide (MTT) assay

Cell viability was measured using the MTT assay (Sigma, St. Louis, MO, USA). The cells (2 × 10^4^ cells/ml) were cultured in 96-well plates in complete medium for 24 h. Then, the medium was replaced with fresh medium containing 0.5% FBS, and the cells were given different treatments. After the treatments, 20 μL of MTT, at a concentration of 5 mg/mL, was added, and the cells were incubated for 4 h. Thereafter, the medium was discarded, and 150 μL of DMSO was added for 10 min. Absorbance at 570 nm was measured for each well using an ELISA microplate reader. All assays were performed in triplicate and repeated three times.

### Caspase-3/7 activity assay

Apo-ONE Homogeneous Caspase-3/7 Assay (Promega, Madison, WI, USA) was used to measure apoptosis in the NRVCs. Briefly, the cells were plated in 96-well plates at a density of 10^4^ cells/well, and were given different treatments. Then, 100 μL of Apo-ONE Homogeneous Caspase-3/7 Reagent was added to each well. The plate was incubated for 1 h at room temperature, using a plate shaker, at 350 rpm. Subsequently, fluorescence was measured using a fluorometer with excitation at 499 nm and emission at 521 nm. All assays were performed in triplicate and repeated three times.

### CaMKII activity assay

The CAMKII Elisa kit (BlueGene) was used to measure CaMKII activity in NRVCs. Briefly, the cells were given different treatments and lysed. Then 100 μL of standards or samples were added to the antibody pre-coated Microtiter Plate, with 100 μl PBS added to the blank control well. We dispensed 10 μl of balance solution, only into samples, and mixed it well. Then we added 50 μl of Conjugate to each well (not the blank control wells) and mixed it well. Then the plate was incubated for 1 h at 37 °C. After washing five times, we added 50 μL Substrate A and B to each well, including the blank control well. The wells were covered and incubated for 10–15 min at 37 °C. Finally, we added 50 μL of stop solution to each well, including the blank control well. We determined the OD at 450 nm using a microplate reader immediately. All assays were performed in triplicate and repeated three times.

### Immunoblotting

After rinsing in cold PBS three times, the cells were homogenized in RIPA buffer. The supernatant was then centrifuged at 120,000×*g* for 15 min at 4 °C. The samples (10–20 mg) were run on SDS-PAGE gels, transferred to PVDF filter membranes, and used for western blotting with monoclonal antibodies against CaMKIIδ (Santa Cruz Biotechnology, Santa Cruz, CA, USA), p47^phox^, Nox2/gp91^phox^ (Abcam, Cambridge, MA, USA), cleaved caspase-3 (Asp175) (Cell Signaling Technology), and ApoJ (EterLife, Birmingham, UK). The PVDF membranes were then incubated with HRP-conjugated anti-rabbit immunoglobulin G antibody (Santa Cruz Biotechnology, Santa Cruz, CA, USA) for 1 h. The blot was developed with an ECL-Plus chemiluminescence reagent kit and visualized with the UVP Bio-Imaging System. The blot densities were analyzed using Image J.

### Statistics

All values in the text and figures are presented as means ± SD of n independent experiments. All data (except western blotting density) were fed to ANOVA, followed by a Bonferroni correction for post hoc tests. Western blot densities were analyzed with the Kruskal–Wallis test, followed by Dunn’s post hoc tests. *P* < 0.05 was considered statistically significant.

## Results

### ApoJ overexpression prevented cell injury induced by ox-LDL

**Fig. 1:**
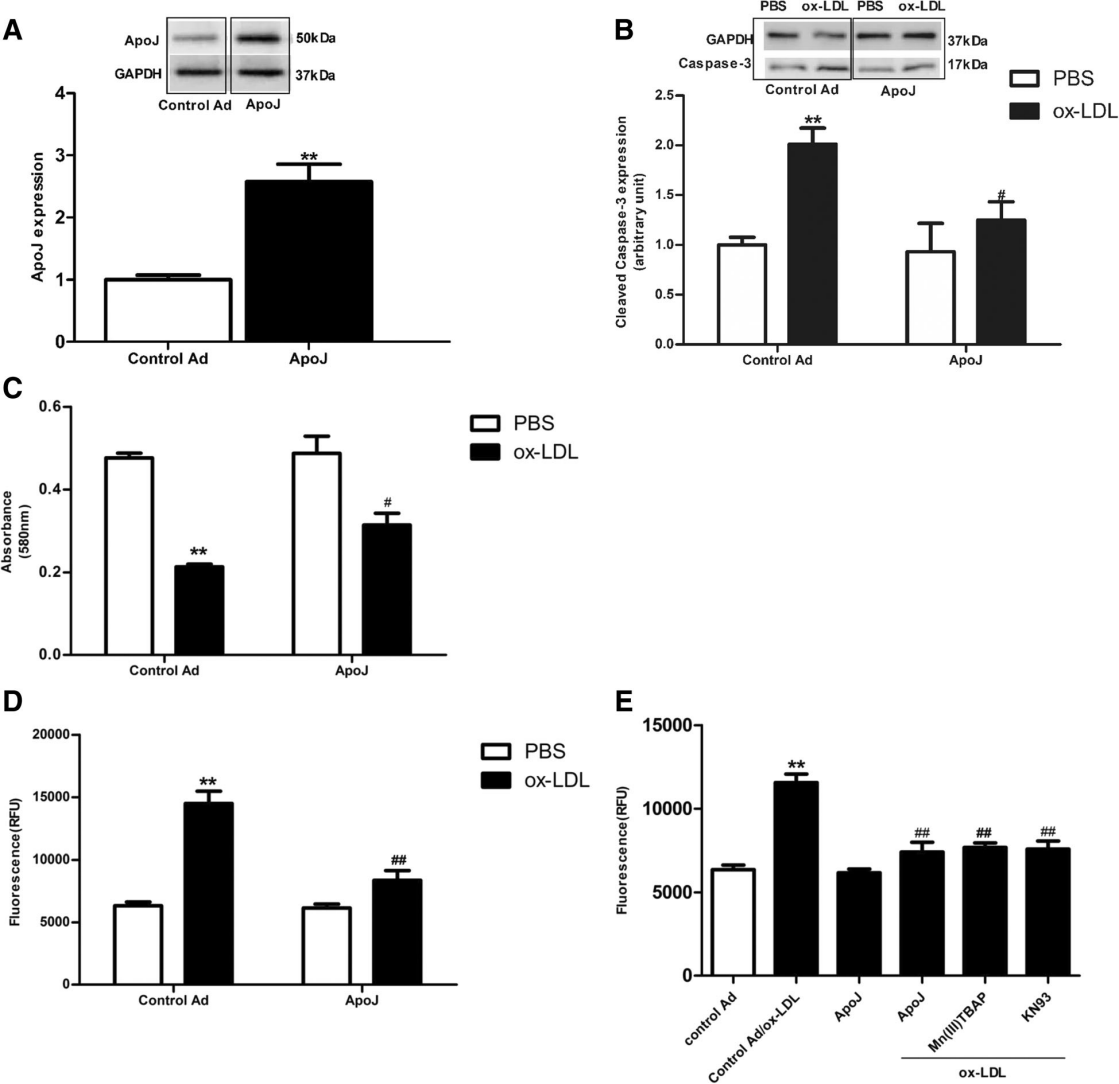
ApoJ overexpression attenuated ox-LDL-induced apoptosis in NRVCs. **a**. ApoJ expression, after infection by recombinant adenovirus, as determined by western blotting (*n* = 4/group). **b**. Cleavage of caspase-3 was enhanced by ox-LDL, but was prevented by ox-LDL (*n* = 4/group). **c**. Ox-LDL reduced cell viability, but ApoJ, as determined by MTT assay (*n* = 8/group), increased it. **d**. Cardiomyocyte apoptosis as determined by caspase-3/7 activity assay. Apoptosis significantly increased, while ApoJ resisted ox-LDL-induced NRVCs injury (n = 8/group). **e**. Cell apoptosis as determined by caspase-3/7 activity assay. Apoptosis of control adenovirus-infected cells increased markedly, while cells pretreated with Mn (III) TBAP and KN93 repelled ox-LDL’s detrimental effect (*n* = 8/group). **P < 0.01 versus control Ad/PBS group; ^#^*P* < 0.05 versus control Ad/ox-LDL group, ^##^*P* < 0.01 versus control Ad/ox-LDL group

To determine whether ApoJ attenuated cell injury induced by ox-LDL, the NRVCs were infected with recombinant adenovirus with and without ApoJ. Compared to the control adenovirus-infected cells, ApoJ expression increased significantly in the Ad-ApoJ-infected NRVCs (Fig. 1a). Next, we analyzed the effect of ApoJ in relation to ox-LDL-induced cell injury. Ox-LDL was added into the NRVCs 36 h after adenovirus infection. As shown in Fig. 1b, ox-LDL administration enhanced cleaved caspase-3 expression, which was markedly attenuated by ApoJ overexpression, as evidenced by the immunoblotting assay. Similar results are seen in Fig. 1c and d—exposure to ox-LDL significantly decreased cell viability, while ApoJ overexpression markedly prevented decreased cell viability induced by ox-LDL (Fig. 1c). Furthermore, ox-LDL increased the percentage of apoptotic cardiomyocytes, which were markedly reduced by ApoJ overexpression (Fig. 1d). These results suggest that ApoJ conferred prominent resistance to cell injury induced by ox-LDL.

Then, we analyzed the ox-LDL downstream inducers, which might mediate cell injury and might be mediated by ApoJ to prevent ox-LDL induced-injury. We used a CAMKII inhibitor [KN93] and a ROS scavenger [Mn (III)TABP] [7] in the presence of ox-LDL. As shown in Fig. 1e, Mn (III) TBAP and KN93 markedly decreased the percentage of apoptotic cells induced by ox-LDL. These results indicate that ox-LDL stimulates cell apoptosis through the CaMKII and ROS pathways.

### ApoJ prevented ROS activation

**Fig. 2:**
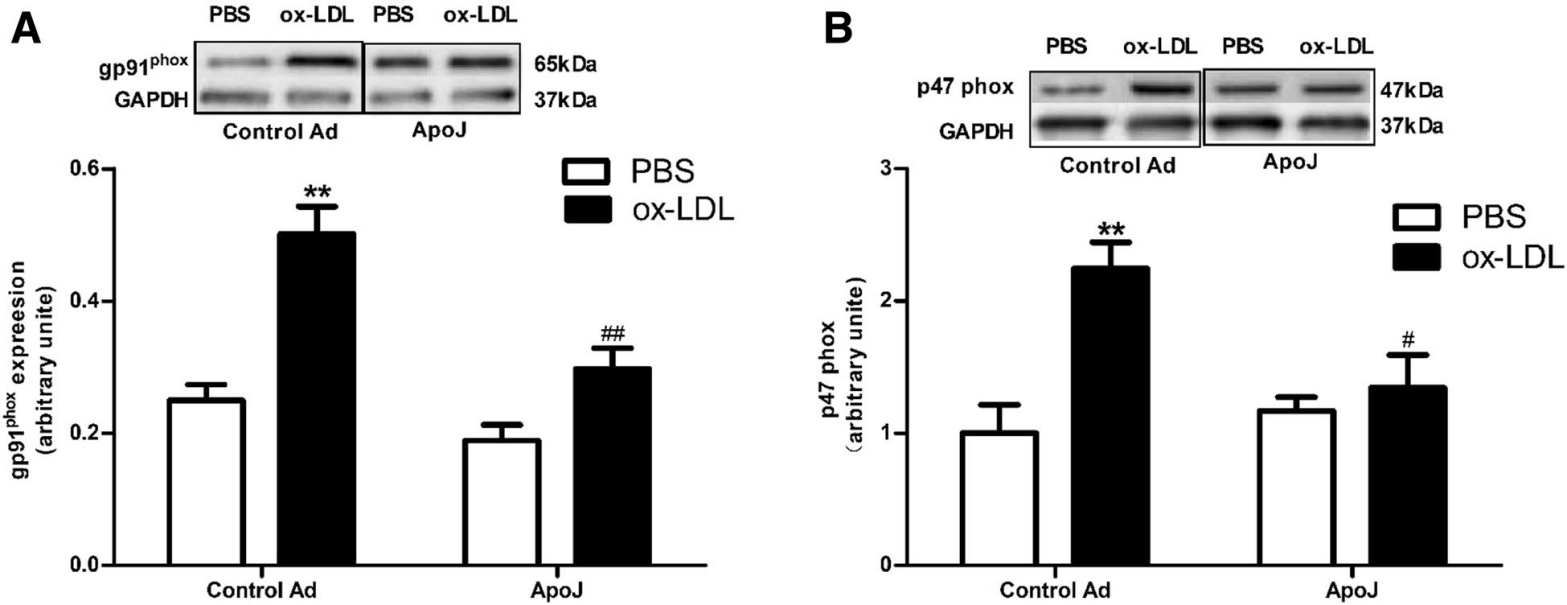
ApoJ attenuated the ox-LDL-stimulated ROS production. **a**. Nox2/gp91^phox^ expression in NRVCs, as determined by western blotting. Ox-LDL enhanced Nox2/gp91^phox^ expression in control adenovirus-infected cells, which was attenuated by ApoJ-overexpression (*n* = 4/group). **b**. Nox2/p47^phox^ expression, as determined by western blotting (*n* = 4/group). Ox-LDL increased Nox2/p47^phox^ expression in control adenovirus-infected NRVCs, while ApoJ overexpression prevented Nox2/p47^phox^ expression. ^*^P < 0.05 versus control Ad/PBS group, ^**^P < 0.01 versus control Ad/PBS group; ^#^*P* < 0.05 versus control Ad/ox-LDL group, ^##^*P* < 0.01 versus control Ad/ox-LDL group

To check whether the antiapoptotic pathway employed by ApoJ involved ROS activation, we analyzed the Nox2/gp91^phox^ expression levels. Ox-LDL induced higher Nox2/gp91^phox^ expression than that in the control adenovirus-infected cells, and the increased Nox2/gp91^phox^ expression was significantly attenuated by ApoJ (Fig. 2a). Similar results are seen in Fig. 2b—p47^phox^ expression, stimulated by ox-LDL administration, while ApoJ attenuated the increased p47^phox^ expression induced by ox-LDL. These results hint at ApoJ-mediated inhibition of ox-LDL-induced ROS production in NRVCs.

### ApoJ overexpression attenuated CaMKII activity and expression caused by ox-LDL

**Fig. 3:**
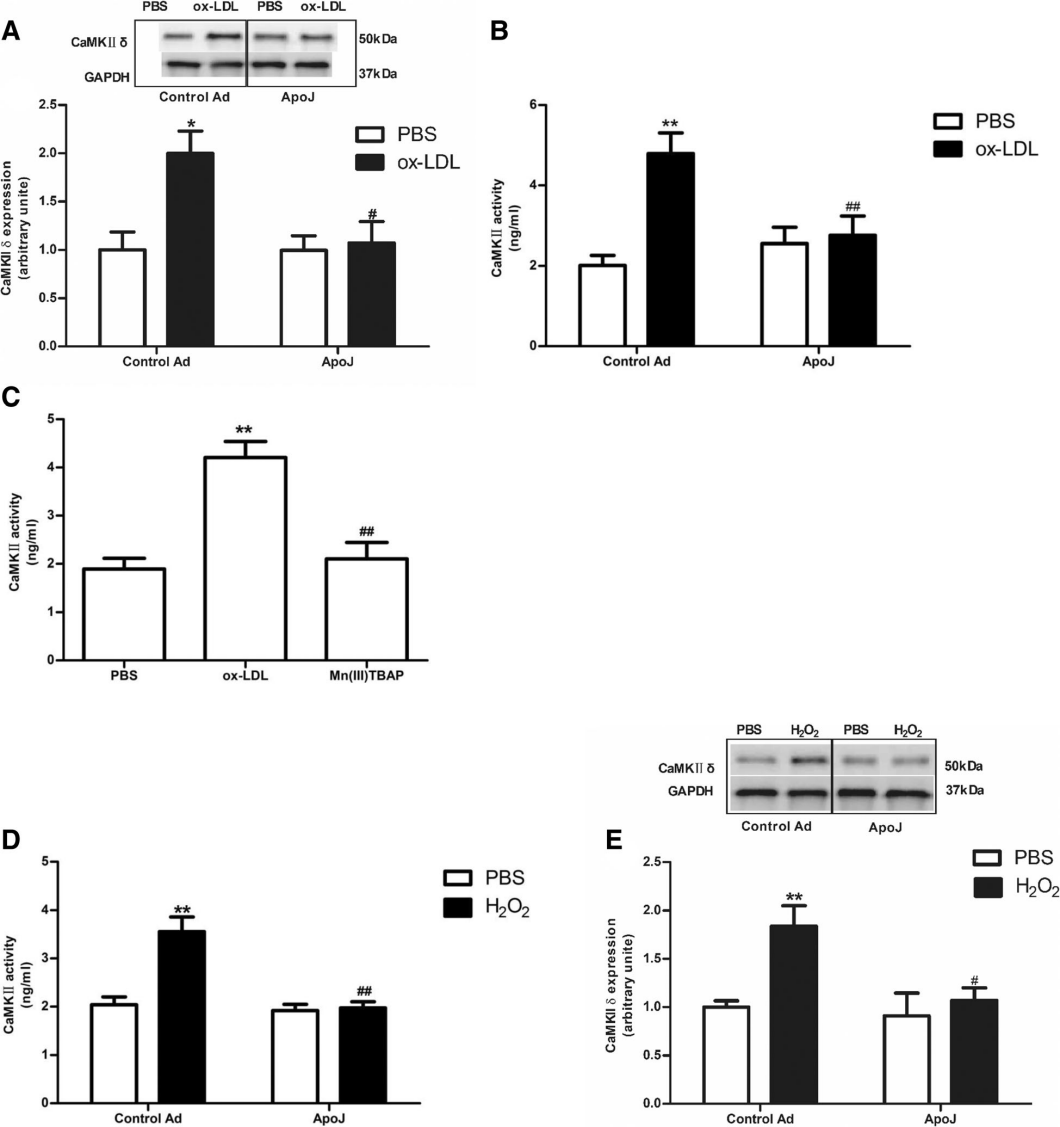
Effects of ApoJ on CaMKII activation in NRVCs. **a**. CaMKIIδ expression in NRVCs. Ox-LDL increased CaMKIIδ expression in control infected cells, while ApoJ overexpression decreased it (*n* = 4/group). **b**. CaMKII activity in NRVCs. Ox-LDL addition enhanced CaMKII activity, while ApoJ overexpression markedly attenuated it (n = 8/group). **c**. CaMKII activity in NRVCs. Mn9(III) TBAP inhibited ox-LDL-enhanced CaMKII activity in NRVCs (*n* = 8/group). **d**. CaMKII activity in NRVCs. H_2_O_2_ addition enhanced CaMKII activity, while ApoJ overexpression markedly attenuated it (*n* = 8/group). **e**. CaMKIIδ expression in NRVCs. H_2_O_2_ increased CaMKIIδ expression in control adenovirus-infected cells, while ApoJ overexpression decreased it (*n* = 4/group). *P < 0.05 versus control Ad/PBS group; **P < 0.01 versus control Ad/PBS group, ^#^*P* < 0.05 versus control Ad/ox-LDL group, ^##^*P* < 0.01 versus control Ad/ox-LDL group. ^#^*P* < 0.05 versus control Ad/ H_2_O_2_ group, ^##^*P* < 0.01 versus control Ad/ H_2_O_2_ group

To investigate whether ApoJ protects NRVCs through the CaMKII pathway, we measured CaMKII activity. As shown in Fig. 3a, ox-LDL administration markedly enhanced CaMKIIδ expression compared to that in the control adenovirus-infected cells, while ApoJ overexpression significantly prevented ox-LDL-stimulated CaMKIIδ expression. Similar results are seen in Fig. 3b; ox-LDL administration increased CaMKII activity, while ApoJ overexpression decreased the CaMKII activity caused by ox-LDL. The increased CaMKII activity, caused by ox-LDL, was attenuated by Mn (III) TBAP, suggesting that ROS may be the upstream activators of CaMKII (Fig. 3c). To further elucidate the underlying pathway responsible for ApoJ’s protective effect on NRVCs, ApoJ-overexpressing cells were treated with exogenous H_2_O_2_, which showed that H_2_O_2_ markedly enhanced CaMKII expression and activity while ApoJ decreased it (Fig. 3d and e).

## Discussion

We thus successfully demonstrated that ApoJ attenuated ox-LDL-induced NRVC apoptosis. In addition, we found that the cardioprotection exerted by ApoJ against ox-LDL may be mediated through the ROS-CaMKII pathway.

Plasma high-density lipoprotein (HDL) levels are inversely proportional to CHD risk. ApoJ is associated with HDL subclasses, implying that ApoJ’s beneficial effect is linked to HDL. ApoJ, a circulating glycoprotein, can be induced by injury such as atherosclerosis and myocardial infarction [8, 9]. Moreover, it has been demonstrated that, in addition to being necessary for cell survival, ApoJ is associated with cell apoptosis and is also expressed in apoptosis-resistant cells [10–14]. Methoxyacetic acid (MAA) administration induces ApoJ located in the cytoplasm, thus exerting protection against apoptotic cell death [15]. ApoJ overexpression protects cells from apoptosis [12, 13], and ApoJ biosynthesis blocks increased cell apoptosis in vitro. ApoJ also delays apoptosis [16] and limits auto-immune autocarditis severity [17]. ApoJ deficiency could worsen brain damage after neonatal hypoxia-ischemia. Therefore, ApoJ may serve as an antioxidative agent, protecting cells from apoptosis caused by ROS [14].

Here, we overexpressed ApoJ in NRVCs to determine whether elevated ApoJ prevented ox-LDL cytotoxicity and attempted to unravel the downstream activator responsible for these effects. We found that ApoJ overexpression increased cell viability and decreased apoptosis compared to the control cells. Moreover, we found that CaMKII inhibitors and ROS scavengers exerted cardioprotective effects on NRVCs in the presence of ox-LDL, similar to the effects afforded by ApoJ. This suggested that CaMKII and ROS may participate in the signaling pathways regulating ApoJ’s effects on cell injury induced by ox-LDL.

Apoptosis occurs in many processes through multiple pathways [18]. Gp91phox was the membrane subunit and p47phox was the cytosolic subunit associated with NOX2, which was a subunit of the NADPH oxidase. It has been reported that increased expression of gp91phox and p47phox contribute to ROS overproduction [19, 20]. Enhanced ROS production could cause cell apoptosis, thus initiating pathological processes. Jun et al. [21] discovered that ApoJ significantly attenuated H_2_O_2_-induced cardiomyocyte apoptosis. ApoJ has also been reported to decrease intracellular ROS levels in human corneal endothelial cells and retinal pigment epithelial cells [22, 23]. We have previously shown that adenovirus-mediated ApoJ overexpression abrogates the elevated ROS production induced by AngII, as determined by reduced Nox2/gp91^phox^ expression in NRVCs. This indicates downstream ROS pathways may take part in ApoJ’s antiapoptotic mechanisms. In the present study, we discovered ApoJ overexpression, mediated by the adenovirus, attenuated the increased ROS production caused by ox-LDL, as evidenced by decreased Nox2/gp91^phox^ and P47^phox^ expression in the NRVCs. These results suggest that ROS is a downstream agent regulating ApoJ’s antiapoptotic effects caused by ox-LDL. However, the underlying mechanisms by which the elevated ROS cause the detrimental effects remain largely unknown.

Calcium (Ca2+)/calmodulin (CaM)-dependent protein kinase II (CaMKII) may serve as a ROS sensor in the heart [24]. CaMKII is now considered a key enzyme in cardiac disease pathologies, such as myocardial infarction, heart failure, and malignant arrhythmias [25, 26]. There are four main posttranslational modifications that regulate the CaMKII activity maintenance independent of the Ca2+/calmodulin-binding—autophosphorylation [27, 28], oxidation [25, 29, 30], O-linked N-acetylglucosamination [31], and S-nitrosylation [32]. CaMKII is ubiquitously expressed and acts as a multifunctional protein kinase with four isoforms, α, β, γ, and δ. α and β isoforms are predominantly expressed in the brain, where they play a critical role in neuronal function. γ and δ isoforms are mainly expressed in the heart. Cardiac CaMKII expression and activity are increased in CHD, and elevated CaMKII activity augments CHD severity.

In vivo and in vitro studies have demonstrated that sustained CaMKII activation induces cell death, and CaMKII inhibition relieves cell injury induced by processes such as endoplasmic reticulum stress and oxidative stress. In our study, we found that ox-LDL significantly enhanced CaMKII expression and activity in NRVCs, which were abrogated by ApoJ overexpression. Furthermore, co-treatment with a CaMKII inhibitor or ROS scavenger prevented ox-LDL-elicited CaMKII activity and expression, suggesting that ROS may be upstream activators of CaMKII in terms of regulating NRVC apoptosis caused by ox-LDL. To further analyze the role of ROS in this process, we treated NRVCs with H_2_O_2_. Our results showed that CaMKII activity and CaMKIIδ expression were markedly augmented in contrast to the control adenovirus-infected cells. In addition, H_2_O_2_-induced CaMKII activation was prevented by ApoJ overexpression.

## Conclusions

Increased ROS production, caused by ox-LDL-induced CaMKII activation, may occur through posttranslational oxidation modification, and ApoJ exerts its cardioprotection by inhibiting the ROS-CaMKII pathway.

### Study limitations

ApoJ contains an antiparallel, disulfide-linked heterodimer comprising an α-chain and a β-chain. The α- and β-subunits are linked by five closely spaced disulfide bonds, enabling the protein to self-associate and form higher oligomers to further improve protein homeostasis. However, those disulfide bonds may be not adequately established in the overexpressed ApoJ, Those cysteines not involved in disulfide bonds may act as ROS scavengers [33]. In this case, the function of ApoJ is compromised. We intend to further investigate the disulfide bond’s stability in the future.

## Abbreviations

ApoJ: Apolipoprotein-J
CaMKII: Calcium (Ca2+)/calmodulin (CaM)-dependent protein kinase II
CHD: Cardiovascular heart disease
HDL: High density lipoproteins
NRVCs: Neonatal rat ventricular cells
ox-LDL: Oxidized low-density lipoprotein
ROS: Reactive oxygen species
SD: Sprague-Dawley
